# Subclinical inflammation associated with prolonged TIMP-1 upregulation and arterial stiffness after gestational diabetes mellitus: a hospital-based cohort study

**DOI:** 10.1186/s12933-017-0530-x

**Published:** 2017-04-13

**Authors:** Tiina Vilmi-Kerälä, Anneli Lauhio, Taina Tervahartiala, Outi Palomäki, Jukka Uotila, Timo Sorsa, Ari Palomäki

**Affiliations:** 1 School of Medicine, University of Tampere, Tampere, Finland; 2 Department of Obstetrics and Gynecology, Tampere University Hospital, Box 2000, 33521 Tampere, Finland; 3 Department of Infectious Diseases, Inflammation Center, Helsinki University Hospital, Helsinki, Finland; 4 Clinicum, University of Helsinki, Helsinki, Finland; 5 The Social Insurance Institution of Finland, Benefit Services, Helsinki, Finland; 6 Department of Oral and Maxillofacial Diseases, Helsinki University and University Hospital, Helsinki, Finland; 7 Division of Periodontology, Department of Dental Medicine, Karolinska Institutet, Huddinge, Sweden; 8 Department of Emergency Medicine, Kanta-Häme Central Hospital, Hämeenlinna, Finland

**Keywords:** Arterial compliance, Gestational diabetes mellitus, High-sensitivity C-reactive protein, Matrix metalloproteinase-8, Matrix metalloproteinase-9, Pulse wave velocity, Subclinical inflammation, Tissue inhibitor of matrix metalloproteinase-1

## Abstract

**Background:**

Gestational diabetes mellitus (GDM) has significant implications for the future health of the mother. Some clinical studies have suggested subclinical inflammation and vascular dysfunction after GDM. We aimed to study whether concentrations of high-sensitivity C-reactive protein (hsCRP), tissue inhibitor of metalloproteinase-1 (TIMP-1), matrix metalloproteinase-8 (MMP-8) and -9, as well as values of arterial stiffness differ between women with and without a history of GDM a few years after delivery. We also investigated possible effects of obesity on the results.

**Methods:**

We studied two cohorts—120 women with a history of GDM and 120 controls—on average 3.7 years after delivery. Serum concentrations of hsCRP were determined by immunonephelometric and immunoturbidimetric methods, MMP-8 by immunofluorometric assay, and MMP-9 and TIMP-1 by enzyme-linked immunosorbent assays. Pulse wave velocity (PWV) was determined using the foot-to-foot velocity method from carotid and femoral waveforms by using a SphygmoCor device. Arterial compliance was measured non-invasively by an HDI/PulseWave™CR-2000 arterial tonometer. All 240 women were also included in subgroup analyses to study the effect of obesity on the results. Multiple linear regression analyses were performed with adjustment for confounding factors.

**Results:**

PWV after pregnancy complicated by GDM was significantly higher than after normal pregnancy, 6.44 ± 0.83 (SD) vs. 6.17 ± 0.74 m/s (p = 0.009). Previous GDM was also one of the significant determinants of PWV in multiple linear regression analyses. On the other hand, compliance indices of both large (p = 0.092) and small (p = 0.681) arteries did not differ between the study cohorts. Serum TIMP-1 levels were significantly increased after previous GDM (p = 0.020). However, no differences were found in the serum levels of MMP-8, MMP-9 or hsCRP. In subgroup analyses, there were significantly higher concentrations of hsCRP (p = 0.015) and higher PWV (p < 0.001) among obese women compared with non-obese ones.

**Conclusions:**

PWV values were significantly higher after GDM compared with normoglycemic pregnancies and were associated with prolonged TIMP-1 upregulation. Cardiovascular risk factors were more common in participants with high BMI than in those with previous GDM.

## Background

In developed countries, the prevalence of gestational diabetes mellitus (GDM) has increased rapidly in recent decades, along with increasing rates of obesity [1, 2]. In Finland, GDM complicated 15.9% of pregnancies in 2015 [2]. A diagnosis of GDM has significant implications for the future health of the mother. For instance, GDM has been shown to be associated with postpartum insulin resistance, hypertension, and dyslipidemia [3–5], placing affected women at risk of metabolic syndrome (MetS), type 2 diabetes mellitus (T2DM) and/or cardiovascular disease (CVD) later in life [5–8]. Incidence of CVD events, and specifically those of coronary artery disease, is known to be increased in women with previous GDM, even in the absence of T2DM [8]. Clinical studies have also revealed subclinical inflammation and vascular dysfunction after GDM [4].

High-sensitivity C-reactive protein (hsCRP) is a well-known acute-phase protein and a sensitive biomarker of systemic inflammation. Elevated levels of hsCRP are a significant risk factor for atherosclerosis [9]. The group of matrix metalloproteinases (MMPs) comprises over 20 structurally and functionally related but genetically distinct members [10, 11]. Expression and activity are normally low, but increased in many pathophysiological conditions. MMPs can modulate immunological responses, and MMPs can be either defensive or destructive [11]. Both upregulation and down-regulation of MMP-8 and -9 have been associated with several noninfectious as well infectious inflammatory states [12–18]. MMP-8 may also regulate blood pressure [19]. MMPs and their inhibitors, tissue inhibitors of MMPs (TIMPs) have been related to atherosclerosis development and progression in humans [20–22]. It has been suggested that imbalanced concentrations of MMP family members and TIMPs eventually exert an important role in cardiovascular risk [21–25].

Inflammation may be pathogenic, by inducing vascular dysfunction [4, 26]. Arterial stiffness has proven to be an important parameter for the assessment of cardiovascular risk, and it has earlier been associated with endothelial dysfunction [27, 28]. Carotid to femoral pulse wave velocity (PWV) has emerged as the gold standard to assess arterial stiffness [29]. When the arteries are stiff or less distensible, PWV increases [30, 31]. PWV increases proportionally to the number of cardiovascular risk factors present, such as diabetes or MetS [27, 32, 33]. In epidemiological studies, increased PWV has been predictive of cardiovascular events [29].

Recently, the implications of GDM as regards women’s future health have been widely discussed. As the prevalence of GDM has increased over the years, a better understanding of the connections between previous GDM and both subclinical inflammation and vascular dysfunction would be of great benefit. In addition, recently it has been suggested that MMP-8 is associated with insulin receptor degradation, and high serum MMP-8 levels with an increased risk of diabetes mellitus type II [17]. In previous studies serum levels of MMP-8, -9, TIMP-1 and hsCRP have been shown to be biomarkers reflecting low-grade inflammation [11, 23, 24, 34, 35]. In addition, TIMP-1 has been shown to exert MMP-independent actions such as pro-inflammatory and growth-factor-like properties [36–38].

With this background our aim was to define whether or not cardiovascular risk, assessed by serum concentrations of hsCRP, MMP-8, MMP-9 and TIMP-1, and values of arterial compliance and PWV are enhanced already a few years after GDM. We also evaluated the effect of obesity on the results.

## Methods

In this follow-up study of two cohorts, a total of 120 women with a history of GDM during the index pregnancy were compared with 120 age-matched women with normal glucose metabolism during pregnancy. The time from the index pregnancy to the follow-up study was also matched between the study groups. All participants had delivered on average 3.7 (range 2–6) years earlier at Kanta-Häme Central Hospital, Finland, i.e. after the publication of Finnish Current Guidelines for screening GDM. Our national guidelines were published in 2008 and updated in 2013 without any change in the diagnostic criteria of GDM [39]. The complete inclusion and exclusion criteria, with power analysis, have been described earlier [40]. Briefly, GDM was defined (using the diagnostic criteria of Finnish Current Guidelines) as a pathological value in a 2-h 75-g oral glucose tolerance test (OGTT) during pregnancy: venous plasma glucose ≥5.3 mmol/L when fasting, ≥10.0 mmol/L at 1 h or ≥8.6 mmol/L at 2 h [39]. Our national diagnostic thresholds for GDM are similar to those of the International Association of Diabetes and Pregnancy Study Groups (IADPSG): plasma glucose ≥5.1 mmol/L when fasting, ≥10.0 mmol/L at 1 h or ≥8.5 mmol/L at 2 h [41]. Only singleton pregnancies were included. Women were excluded if they had type 1 or type 2 diabetes before the pregnancy, if they were pregnant at time of the study, if they had suspected or verified malignant or endocrine disease, if there was substance abuse or treatment, or a known clinical history of psychiatric illness. Controls had to have normal OGTT results during pregnancy. If the controls had experienced GDM in an earlier pregnancy, or the weight of the newborn was ≥4.5 kg, they were excluded. The electronic database of the hospital was used to pick up the cases and controls. Both recruitment and examinations were accomplished between August 2011 and July 2014.

We interviewed the participants as regards their lifestyle habits. Lifetime tobacco exposure was estimated as pack-years, and one pack-year was defined as 20 cigarettes smoked every day for 1 year [42]. Further, we interviewed the participants as regards their history of trauma or infectious diseases during the previous month. We measured resting heart rate, brachial blood pressure, weight (kg) and height (cm) of the participants, and calculated body mass index (BMI): weight in kilograms divided by height in meters squared (kg/m^2^).

The study was conducted in accordance with the ethical principles outlined in the Declaration of Helsinki [43], and the protocol was approved by the Ethics Committee of Kanta-Häme Hospital District (reference number 521/2010; date of approval 21.12.2010). Every participant was given both oral and written information on the study before she signed an informed consent document.

### Laboratory methods

Serum samples were collected after at least 12 h of fasting and stored at −80 °C until analyzed. Serum concentrations of hsCRP were analyzed according to validated immunonephelometric (United Medix Laboratories Ltd., Espoo, Finland) and immunoturbidimetric (VITA Healthcare Services Ltd., Vita Laboratory, Helsinki, Finland) methods [44, 45]. Concentrations of MMP-8 were determined by immunofluorometric assay (IFMA) (Medix Biochemica, Espoo, Finland), as previously described [25]. Serum levels of MMP-9 and TIMP-1 were analyzed by enzyme-linked immunosorbent assay (ELISA) using commercial kits (Biotrak ELISA System; Amersham Biosciences, GE Healthcare, Buckinghamshire, UK) and according to the manufacturer’s instructions [18]. Fasting serum levels of total cholesterol (TC) and insulin were analyzed according to validated methods as described in detail earlier [40].

### Determination of arterial compliance and pulse wave velocity

Three experienced nurses measured the compliance of large and small arteries after at least 10 min of rest in a semi-sitting position. The recording was carried out after an overnight fast. The participants were asked to refrain from eating, having caffeinated drinks, smoking and taking medication for 12 h, and drinking alcohol for 2 days prior to measurement. Radial artery pulse waves were recorded non-invasively with an arterial tonometer (HDI/PulseWave™CR-2000, Hypertension Diagnostics, Inc., Eagan, Minnesota, USA) and the procedure involves the use of a modified Windkessel pulse-contour method [46]. Blood volume inertia and systemic vascular resistance are used to analyze arterial compliance. The capacitive compliance of large arteries (C1), including the aorta, and the endothelial function of small arteries (C2) were automatically assessed as a mean of the five most similar pulse waves appearing during 30-s of measurement. Three consecutive measurements were performed to obtain mean results for every participant.

Carotid-femoral PWV was measured using the foot-to-foot velocity method from carotid and femoral waveforms by employing a SphygmoCor device (AtCor Medical, Sydney, Australia). Transcutaneous readings were obtained at the right common carotid artery and the right femoral artery with the subjects in a supine position with direct-contact pulse sensors. The time delay (Dt or transit time) of the two waveforms was registered, and the distance (D) between carotid and femoral recording sites was obtained by subtracting the carotid measurement site to sternal notch distance from the sternal notch to the femoral measurement site distance. PWV was calculated as follows: D/Dt (m/s) [29, 30]. Three measurements were performed to obtain average results for every participant. Only measurements that met the automatic quality control cutoff were used in the final analysis. All the PWV measurements were performed by two experienced nurses.

### Statistical analysis

The data were analyzed by using IBM^®^ SPSS^®^ Statistics Version 23 software (copyright 2015). Variables were tested for normality by way of Shapiro–Wilk or Kolmogorov–Smirnov tests, as appropriate. Data are presented as mean ± standard deviation (SD) if not mentioned otherwise. Differences in continuous variables between GDM participants and controls were studied by using Student’s *t* test in cases of normality and the Mann–Whitney *U* test in cases of skewed distribution of measurements.

All 240 women were also included in subgroup analyses to study the effect of obesity on the results. For these analyses, we divided the whole study group into four subgroups according to obesity and previous GDM. Obesity was classified as BMI ≥30 kg/m^2^ [47]. The clinical characteristics of these four subgroups were studied by way of one-way ANOVA in cases of normality and by using the Kruskal–Wallis test in cases of non-normality. If the overall *p* value was significant, individual *p* values between subgroups were also calculated. Post hoc analyses, with a conservative Bonferroni correction factor, were performed in order to correct for multiple testing. The relationships between different cardiovascular risk factors were tested by Pearson’s or Spearman’s correlation analysis, as appropriate.

Further, we conducted univariate linear regression analyses for hsCRP, MMP-8, TIMP-1, PWV and arterial compliance index values to find possible associations with clinically relevant covariates. Then multivariable linear analyses were carried out to examine whether simple associations were changed after adjustment for potential confounders. Finally, stepwise multiple linear regression analyses were done to find out relevant covariates to final models. The selected covariates in all of these analyses were age, BMI, previous GDM, time after the index pregnancy, pack-years of smoking, heart rate, systolic blood pressure, hsCRP, TC and fasting insulin. F-statistics was used to optimize the sequential variable selection procedure. A two-tailed probability value of <0.05 was considered significant.

## Results

The basic clinical characteristics of the study participants are summarized in Table 1. There were no significant differences between the two cohorts in self-reported history of respiratory infection, other infectious disease or trauma during the month before follow-up laboratory examinations.

**Table 1:** Basic clinical characteristics of women with GDM and controls

	GDM	Controls	*p* value
Average time since delivery, years	3.7 ± 1.0	3.7 ± 0.9	0.818
Age, years	35.8 ± 4.4	35.9 ± 4.6	0.854
Primiparous, n (%)	23 (19.2%)	23 (19.2%)	1.000
Therapy of GDM during pregnancy			
Insulin, n (%)	24 (20.0%)		
Metformin, n (%)	1 (0.8%)		
Dietary therapy, n (%)	95 (79.2%)		
Pack-years of smoking	3.8 ± 6.0	2.4 ± 4.6	0.012
During the previous month, history of			
Respiratory infection, n (%)	45 (37.5%)	44 (36.7%)	0.854
Other infectious disease, n (%)	18 (15.0%)	10 (8.3%)	0.053
Trauma, n (%)	9 (7.5%)	5 (4.2%)	0.264
BMI, kg/m^2^	28.3 ± 5.0	27.5 ± 5.4	0.069
Systolic BP, mmHg	122.4 ± 12.5	119.0 ± 11.5	0.034
Diastolic BP, mmHg	73.5 ± 9.0	71.8 ± 8.7	0.176
Heart rate, beats per minute	65.9 ± 9.1	63.8 ± 9.6	0.017
TC, mmol/L	4.7 ± 0.9	4.6 ± 0.8	0.329
F-Gluc, mmol/L	5.6 ± 0.6	5.3 ± 0.3	<0.001
F-Insu, mU/L	5.2 ± 3.6	4.6 ± 3.6	0.087

Data are presented as mean ± SD if not mentioned otherwise

*BMI* body mass index, *BP* blood pressure, *F-Gluc* fasting glucose, *F-Insu* fasting insulin, *TC* total cholesterol

### Subclinical inflammation

Serum TIMP-1 levels were significantly increased after previous GDM (Table 2). There was a significant positive association between previous GDM and TIMP-1 levels in both univariate and multivariable linear regression analyses (data not shown). There were no differences in the concentrations of MMP-8 and MMP-9 between the groups (Table 2). In stepwise multiple linear regression analyses, hsCRP, previous GDM and TC were important determinants of MMP-8 levels. Likewise, previous GDM, together with BMI and heart rate associated with TIMP-1 in stepwise multiple linear regression analyses. Nevertheless, the significant determinants explained only 13.8% of MMP-8 and 6.7% of TIMP-1 concentrations (Table 3).

**Table 2:** Results of primary analyses of GDM and control groups

	GDM	Controls	*p* value
hsCRP, mg/L	2.50 ± 3.69	2.50 ± 4.19	0.582
MMP-8, ng/mL	27.83 ± 1.48	32.78 ± 1.90	0.082
MMP-9, ng/mL	384.27 ± 13.15	392.15 ± 12.60	0.667
TIMP-1, ng/mL	102.80 ± 29.72	94.58 ± 24.51	0.020
MMP-8/TIMP-1, mol ratio	0.13 ± 0.009	0.17 ± 0.015	0.035
MMP-9/TIMP-1, mol ratio	1.32 ± 0.078	1.43 ± 0.085	0.152
C1, mL/mmHg × 10	15.14 ± 3.51	15.85 ± 3.36	0.092
C2, mL/mmHg × 100	8.44 ± 3.08	8.60 ± 3.20	0.681
PWV, m/s	6.44 ± 0.83	6.17 ± 0.74	0.009

Data are presented as mean ± SD

*hsCRP* high-sensitivity C reactive protein, *C1* large artery compliance index, *C2* small artery compliance index, *PWV* pulse wave velocity, *MMP-8* matrix metalloproteinase-8, *MMP-9* matrix metalloproteinase-9

**Table 3:** Results of stepwise multiple linear regression analyses

Parameters	Covariates included in the model	R^2^ for model	Global *p*	Standardized β	*p* value
hsCRP		0.096	<0.001		
	BMI			0.259	<0.001
MMP-8		0.138	<0.001		
	hsCRP			0.312	<0.001
	Previous GDM			−0.137	0.025
	TC			0.129	0.036
TIMP-1		0.067	0.003		
	Previous GDM			0.157	0.015
	BMI			0.149	0.025
	Heart rate			−0.132	0.044
C1		0.524	<0.001		
	Systolic BP			−0.602	<0.001
	Heart rate			−0.347	<0.001
	BMI			0.232	<0.001
	Time after the index pregnancy			−0.095	0.041
C2		0.317	<0.001		
	Systolic BP			−0.345	<0.001
	Heart rate			−0.312	<0.001
	BMI			0.286	<0.001
	Age			−0.191	0.001
	Pack-years of smoking			−0.144	0.012
PWV		0.470	<0.001		
	Systolic BP			0.534	<0.001
	Age			0.230	<0.001
	F-Insu			0.191	<0.001
	Previous GDM			0.105	0.026
	Time after the index pregnancy			−0.102	0.040

Covariates in these analyses included age, BMI, previous GDM, pack-years of smoking, time after the index pregnancy, heart rate, systolic blood pressure, hsCRP, TC and fasting insulin. Final models include significant covariates only. Standardized β provides a measure of the relative strength of an association, independent of the measurement units. Standardized β and *p* values are shown only when *p* < 0.05

*BMI* body mass index, *BP* blood pressure, *F-Insu* fasting insulin, *GDM* gestational diabetes mellitus, *hsCRP* high-sensitivity C reactive protein, *C1* large artery compliance index, *C2* small artery compliance index, *PWV* pulse wave velocity, *MMP-8* matrix metalloproteinase-8

We found no difference in the concentrations of hsCRP between GDM cases and controls (Table 2), even when participants affected with infections or traumas were excluded (data not shown). In stepwise multiple linear regression analysis (Table 3), only BMI was a significant determinant of hsCRP levels, but the model explained only 9.6% of hsCRP values. Previous GDM did not influence hsCRP concentrations in our data.

### Pulse wave velocity and arterial compliance

PWV values differed significantly between the GDM cases and controls (Table 2). In univariate linear regression analysis, there were significant associations with age (p < 0.001), fasting insulin (p < 0.001), previous GDM (p = 0.009), TC (p < 0.001), heart rate (p < 0.001), systolic blood pressure (p < 0.001) and BMI (p < 0.001). In stepwise multiple linear regression analysis, significant determinants of PWV values were systolic BP, age, insulin levels, previous GDM and time after the index pregnancy. Covariates explained 47.0% of PWV (Table 3). In our two study cohorts, there were no interactions between previous GDM and TIMP1 on PWV (data not shown).

There was a nonsignificant difference in C1 values between the study groups. No difference was revealed in C2 values, either. In univariate linear regression analysis, there was no significant association between C2 and BMI (p = 0.726), but an inverse association between C1 and BMI was significant (p = 0.025). In stepwise multiple linear regression analysis, systolic BP, heart rate, BMI and time after the index pregnancy were significant covariates explaining 52.4% of C1 values. Significant determinants of C2 values were systolic BP, heart rate, BMI, age and pack-years of smoking. These covariates explained 31.7% of C2 values (Table 3).

### Effect of obesity in subgroups

**Fig. 1:**
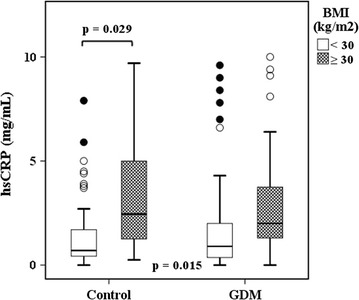
Serum concentrations of hsCRP in the four subgroups. Median values (minimum, maximum) of hsCRP: among obese GDM women 2.1 (0.0, 12.4) mg/mL, obese control women 2.1 (0.3, 18.5) mg/mL, non-obese GDM women 0.9 (0.0, 32.3) mg/mL, and non-obese control women 0.7 (0.0, 25.7) mg/mL. Values of more than 10 mg/mL were measured by turbidimetric immunoassay. The overall *p* value is given at the *bottom*. Individual *p* values for pairwise comparisons are also presented

**Fig. 2:**
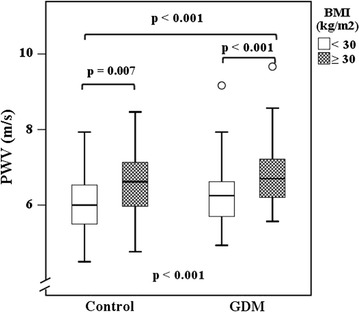
PWV in the four subgroups. Median values (minimum, maximum) of PWV: among obese GDM women 6.8 (5.6, 9.7) m/s, obese control women 6.6 (4.8, 8.5) m/s, non-obese GDM women 6.3 (4.9, 9.2) m/s, and non-obese control women 6.0 (4.5, 7.9) m/s. The overall *p* value is given at the *bottom*. Individual *p* values for pairwise comparisons are also presented

**Fig. 3:**
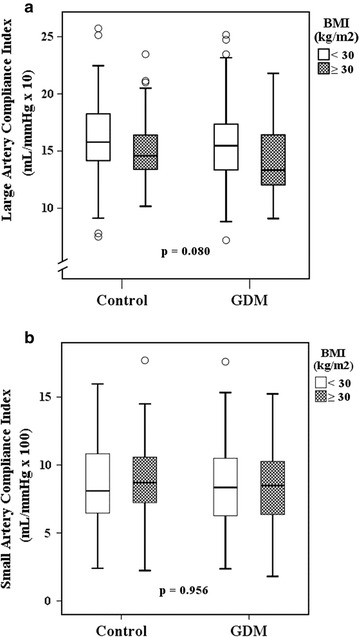
Large (**a**) and small (**b**) artery compliance index values in the four subgroups. **a** Median values (minimum, maximum) of the large-artery compliance index (C1): among obese GDM women 13.3 (9.1, 21.8) mL/mmHg × 10, obese control women 14.7 (10.2, 23.5) mL/mmHg × 10, non-obese GDM women 15.2 (7.2, 25.2) mL/mmHg × 10, and non-obese control women 15.9 (7.5, 25.7) mL/mmHg × 10. The overall *p* value is given. **b** Median values (minimum, maximum) of the small-artery compliance index (C2): among obese GDM women 8.8 (2.8, 15.2) mL/mmHg × 100, obese control women 8.6 (2.2, 17.7) mL/mmHg × 100, non-obese GDM women 8.1 (1.8, 17.6) mL/mmHg × 100, and non-obese control women 8.1 (2.4, 16.0) mL/mmHg × 100. The overall *p* value is given

Altogether, there were 75 women in the obese group (BMI ≥ 30 kg/m^2^); 43 GDM and 32 control participants. The non-obese group (BMI < 30 kg/m^2^; n = 165) consisted of 77 GDM and 88 control participants [55]. In subgroup analyses, participants in obese subgroups had higher serum concentrations of hsCRP than those in non-obese subgroups, as shown in Fig. 1. The concentrations of MMP-8 in the four subgroups were as follows: obese GDM cases, 27.76 ± 1.77 ng/mL, obese controls 37.10 ± 4.16 ng/mL, non-obese GDM cases, 27.88 ± 2.08 ng/mL and non-obese controls, 31.21 ± 2.10 ng/mL. The concentration of MMP-8 was highest among obese controls, but the differences between the four subgroups were not significant (p = 0.090). We also found no differences in the levels of MMP-9 or TIMP-1 between these four subgroups (data not shown). Between the subgroups, there were no differences in the MMP-8/TIMP-1 or MMP-9/TIMP-1 ratio either (data not shown). In the four subgroups, differences in PWV values were significant, but differences in both C1 and C2 values were not (Figs. 2, 3).

## Discussion

Our main finding was that PWV was significantly higher after GDM than after normoglycemic pregnancy. This was supported by a nonsignificant difference in the large-artery compliance index, C1, which indicates that the arteries of GDM cases were less distensible than those of the controls. Secondly, subclinical low-grade inflammation and reduced arterial compliance especially affected women with high BMI.

Inflammation has been shown to be a strong predictor of women’s cardiovascular complications [48]. We found that levels of TIMP-1 were significantly upregulated after previous GDM, reflecting low-grade inflammation among this relatively healthy and young study population. No differences were found in circulating levels of MMP-8 or MMP-9 between the two study cohorts. In subgroup analyses, the highest levels of MMP-8 were in obese controls, but this did not reach statistical significance either. A search of MEDLINE (English language; 1989–September 2016; search terms: “MMP-8, MMP-9, TIMP-1” and “GDM”) revealed no publications concerning female populations where levels of MMP-8, MMP-9 or TIMP-1 have been studied in connection with previous GDM.

There is evidence that glucose can modulate the expression, production and activity of MMPs. For example, endothelial cells cultured in hyperglycemic conditions present increased expression and activity of MMP-9 [49]. It is a pity that there were no samples left for MMP analysis taken from the patients during the period when they suffered from gestational diabetes. We might postulate, that during the pregnancy GDM increase concentrations of MMPs and they in turn upregulate TIMP-1. After the delivery, the decreasing concentrations of glucose, MMPs and TIMP-1 take place consecutively. The prolonged upregulation of TIMP-1 found in this study without upregulated MMP levels may also be a result of the fact that upregulated TIMP-1 may suppress MMP-8 and MMP-9 levels. Further, third explanation for prolonged TIMP-1 upregulation found in this work may be that prolonged elevation of TIMP-1 levels may mediate MMP-independent pro-inflammatory or growth-factor-like signaling functions contributing to low-grade inflammation [36–38].

Recent studies have reported higher CRP and hsCRP levels in women with a history of GDM than in age-matched normal controls after a 1- or 5-year postpartum period [4, 50, 51]. On the contrary, Ajala et al. found no difference in CRP in women after previous GDM compared to controls 4–10 years postpartum [52]. In our study, when hsCRP was determined on average at 3.7 years after delivery, there was no difference between the age-matched study cohorts. However, low-grade inflammation was evident among obese women, in contrast to non-obese participants in subgroup analyses. The GDM and non-GDM women of our study did not differ in BMI, which can partly explain the similar hsCRP levels between the two study cohorts.

Only a few studies have been published concerning a possible relationship between PWV and previous GDM. Lekva et al. reported an enhanced cardiovascular risk at 5-year follow-up as reflected in elevated PWV after previous GDM diagnosed using the old criteria of the World Health Organization (WHO) (OGTT: 2-h plasma glucose ≥7.8 mmol/L). However, they did not find such an association in PWV when using IADPSG diagnostic criteria (OGTT: fasting plasma glucose 5.1–6.9 mmol/L, 1-h plasma glucose ≥10.0 mmol/L or 2-h plasma glucose 8.5–11.0 mmol/L) [41, 53]. Using diagnostic criteria of GDM similar to those of the IADPSG [39], we observed a significant increase in PWV in women with previous GDM. Previous GDM was also a significant determinant of PWV in multiple linear regression analysis. Our results are in accordance with those of Tam et al., who reported higher PWV in women with a history of GDM followed up at a median of 6 years postpartum [54]. In contrast to these findings, Heitritter et al. detected no difference in PWV at an average of 1 year after previous GDM compared with normoglycemic pregnancy [4]. There were no significant differences in C1 or C2 values between the GDM cases and controls. In a recent study, no difference was found in vascular function measured also by using HDI/PulseWave™CR-2000 in women with a history of GDM when compared to healthy controls 4–10 years postpartum, either [52].

Strengths of our study include the fact that we used standardized measurements of arterial stiffness. Determination of systemic arterial stiffness by using HDI/PulseWave™CR-2000 equipment is widely used, and carotid-femoral PWV is accepted as the most reliable measurement of arterial stiffness [29]. We measured the levels of MMP-8, MMP-9 and TIMP-1 by specific immunoassays previously found to be suitable for diagnosis and monitoring of systemic low-grade inflammation associated with cardiovascular and infectious diseases as well as other inflammatory states [11, 13–18, 23–25]. Further, we performed a well characterized hospital-based study of two cohorts of women with a similar follow-up time and age. Moreover, there was no significant difference in BMI between the study groups, and all participants had undergone OGTT screening during the index pregnancy. Since low-risk parturients do not routinely undergo OGTTs in Finland [39], this last strength may also turn out to be a weakness, because the most low-risk women had to be excluded from our study [40]. Although the relatively short time from delivery to the follow-up study allowed us to observe early cardiovascular changes, it may be one of our study limitations as well, since major differences between the study groups are probably better observable later in their life. For example, within 7 years postpartum, previous GDM was identified as a risk factor of CVD by Goueslard et al. They studied database of more than 1.5 million deliveries and found that the incidence of myocardial infarction was 0.04% in women with a history of GDM and 0.02% without [7].

In our subgroup analyses, obesity was associated with higher levels of hsCRP and higher values of PWV. We have earlier revealed the effect of obesity being similar with many other markers for cardio-metabolic risks among the four subgroups [40, 55]. Earlier, BMI has been shown to associate inversely with arterial compliance [56]. As presented in Fig. 3, this seemed to be the case also in our study in C1 values. Surprisingly, in multiple regression analyses, BMI seemed to be protective as regards arterial compliance (C1 and C2). BMI was significantly correlated with systolic blood pressure and heart rate (data not shown). Hence, adjusted findings concerning C1 and C2 might have been affected by these relationships irrespective of possible biologic associations. In our opinion, this result may be explained by multiple interactions of C1 and C2 measurements with other confounding variables. This was supported by the findings of univariate analysis and stepwise multiple linear regression analysis without systolic BP and heart rate as covariates, where inverse association between BMI and C1 was found and association between BMI and C2 was vanished (data not shown).

The prevalence of obesity is increasing around the world [57]. Specifically, visceral obesity modifies glucose and lipid metabolism. It is associated with increased risk of arterial stiffness and atherosclerosis both in normal-weight subjects and patients with T2DM [58, 59]. Our results imply that in preventing cardiovascular risk among women after delivery, we need a comprehensive attitude in clinical care instead of concentrating on single factors.

## Conclusions

When studied 3.7 years after delivery, PWV values were higher in women with previous GDM, indicating that their arteries are less distensible than those in women with previous normoglycemic pregnancy. Among other findings, this relationship was even more evident in obese subjects. We also found that serum levels of TIMP-1 were significantly upregulated after previous GDM, reflecting low-grade inflammation among this relatively healthy and young study population. Altogether, our results demonstrate that previous GDM may reflect a subclinical inflammatory state and together with obesity may contribute to an early stage of the subclinical atherosclerotic process even in relatively young and healthy women.

## Abbreviations

C1: large artery compliance index
C2: small artery compliance index
GDM: gestational diabetes mellitus
hsCRP: high-sensitivity C-reactive protein
MMP: matrix metalloproteinase
OGTT: oral glucose tolerance test
PWV: pulse wave velocity
TIMP: tissue inhibitor of metalloproteinase
